# More Than a Tick-Box: The Importance of the Medicines Reconciliation Process

**DOI:** 10.1192/bjo.2026.11574

**Published:** 2026-06-30

**Authors:** Annabell Agate

**Affiliations:** Sussex Partnership NHS Trust, Eastbourne, United Kingdom

## Abstract

**Aims:**

The Medicines Reconciliation 'Med Rec' is a important, structured process which takes place on admission. It involves comparing the patient’s current medication list from various sources such as the primary care record, previous discharge letters and the patient themselves. This audit aims to highlight the invaluable support of the pharmacy department in ensuring safe prescribing for psychiatric inpatients - a population with a known potential for significant comorbid physical health conditions and high rates of polypharmacy.

NICE guidelines state that “people who are inpatients in an acute setting (including mental health inpatient wards) should have a reconciled list of their medicines within 24 hours of admission.” Sussex Partnership NHS Foundation Trust local targets specify that 60% of Level 2 (pharmacy-led) medicines reconciliations should be completed within 24 hours of admission, or 90% by the next working day by 5pm.

**Methods:**

A total of 100 records were reviewed for patients admitted to Bodiam Ward, an adult male inpatient psychiatric ward in East Sussex, between 2024 and 2025. Of the records reviewed, 73 were retrospective admissions and 27 were prospective records.

**Results:**

In this audit, 66% of medicine reconciliations were completed within 24 hours, and 87% were completed by the next working day. The majority (84%) were completed by Pharmacy Technicians, with the remaining 16% completed by Pharmacists. Of the completed medicine reconciliations, 54% identified at least one omitted drug. The most omitted drug classes were analgesics (13.7%), benzodiazepines (13.1%), and regular oral antipsychotics (10.2%). Other notable omissions included physical health medications, such as cardiovascular drugs (including antihypertensives and statins), and medications related to alcohol dependence (such as thiamine and chlordiazepoxide). In addition, 7% identified ‘other errors’, including missing or incorrect doses, incorrect formulations (immediate-release versus modified-release), and in one instance, an entirely different medication being erroneously prescribed.

**Conclusion:**

This audit demonstrates that medicine reconciliation is more than a 'tick-box exercise' and plays a critical role in identifying prescribing errors on admission. The findings emphasise the significant timely contribution of the pharmacy team to patient safety in adultin patient psychiatric settings. Based on these findings, future education could focus on reminding resident doctors to thoroughly review previous discharge summaries and primary care records when clerking a patient, and to liaise with the pharmacy team for a better standard of care.

